# *Staphylococcus aureus* Induces Goat Endometrial Epithelial Cells Apoptosis via the Autophagy and Endoplasmic Reticulum Stress Pathway

**DOI:** 10.3390/ani12060711

**Published:** 2022-03-11

**Authors:** Yanyan Yi, Kangkang Gao, Ruixue Zhang, Pengfei Lin, Aihua Wang, Yaping Jin

**Affiliations:** Key Laboratory of Animal Biotechnology of the Ministry of Agriculture, College of Veterinary Medicine, Northwest A&F University, Yangling 712100, China; yieryan2015@163.com (Y.Y.); gkk171011@163.com (K.G.); zhangruixue@nwafu.edu.cn (R.Z.); linpengfei@nwsuaf.edu.cn (P.L.); aihuawang1966@163.com (A.W.)

**Keywords:** *Staphylococcus aureus*, goat endometrial epithelial cells, autophagy, ER stress, apoptosis

## Abstract

**Simple Summary:**

The toxicity mechanism of *Staphylococcus aureus* on goat endometrial epithelial cells (gEECs) is still unelucidated. The purpose of this experiment was to investigate the molecular mechanism of gEECs death caused by *S. aureus* in terms of autophagy and endoplasmic reticulum (ER) stress. We found that the accumulation of autophagosomes exacerbated *S. aureus*-induced gEECs apoptosis, and that ER stress was involved in the regulation of the autophagy. These findings may provide new insight into the therapeutic target of endometrial cell injury.

**Abstract:**

Increasing evidence indicates that autophagy and endoplasmic reticulum (ER) stress are involved in the regulation of cell death; however, the role of autophagy and ER stress in *Staphylococcus aureus*-induced endometrial epithelial cell damage is still unelucidated. In the present study, our results showed that infection with *S. aureus* increased the cytotoxicity and the protein expression of Bax, caspase-3, and cleaved-PARP-1 in goat endometrial epithelial cells (gEECs). Moreover, after infection, the expression of LC3II and autophagosomes were markedly increased. The autophagosome inhibitor 3-methyladenine (3-MA) significantly decreased the cytotoxicity and the expression of caspase-3, and cleaved-PARP-1; however, the autophagosome–lysosome fusion inhibitor chloroquine (CQ) increased their expression. Additionally, the protein expression of GRP78, EIF2α, and ATF4 were also markedly increased after infection. The ER stress inhibitor 4-PBA decreased the cytotoxicity and the expression of LC3II and apoptosis-related proteins in *S. aureus*-infected gEECs. Collectively, our findings prove that the accumulation of autophagosomes exacerbated *S. aureus*-induced gEECs apoptosis, and that ER stress was involved in the regulation of the autophagy and apoptosis.

## 1. Introduction

*Staphylococcus aureus* is a recognized zoonotic disease pathogen, leading to a great threat to humans and animals. The structural and functional integrity of the endometrium plays a key role in defending against pathogenic bacterial infections [1]. Therefore, it is necessary to explore the toxicity mechanism of *S. aureus* on the uterus. Endometrial epithelial cells (EECs) are considered to be the first physical line of defense against pathogens that modulate innate immunity and inflammatory responses [2]. Revealing the mechanisms of damage to EECs is essential in order to control the occurrence and establishment of bacterial infections in the uterus. Studies have reported that *S. aureus* promotes nitric oxide release from endothelial and epithelial cells, the inward flow of extracellular Ca^2+^, the production of pro-inflammatory cytokines, the activation of caspase-1 and NLRP3 inflammasome to promote pyroptosis, the cleavage of ligand proteins to disrupt epithelial cell barriers, and the promotion of oxidative stress and apoptosis [3,4,5]. However, the mechanisms of *S. aureus*-induced EECs death still need to be further investigated.

Autophagy is a process by which large intracellular proteins, or even whole cells, are isolated to lysosomes for degradation, which plays a key role in maintaining cellular homeostasis and regulating cell survival and death. Autophagy can be activated by a variety of stressors, including starvation and other intracellular signals, etc. Although, in general, autophagy is a cytoprotective mechanism, excessive autophagy leads to cell death. There is now increasing evidence that many pathogens can induce autophagic cell death in host cells, and that autophagy is involved in the regulation of apoptosis [6,7]. Specifically, it remains unclear whether autophagy was involved in *S. aureus*-induced EECs death.

The endoplasmic reticulum (ER) is a highly dynamic organelle which participates in the regulation of cellular functions, including the control of the lipid metabolism, calcium storage, and protein stabilization. Glucose deprivation, the disruption of calcium homeostasis, and pathogenic infections can disrupt the ER internal environment and impair protein processing and maturation, leading to the accumulation of misfolded proteins and a characteristic stress response known as the unfolded protein response (UPR). The UPR protects cells from stress and contributes to the re-establishment of intracellular homeostasis. However, the UPR promotes cell death under prolonged ER stress. The UPR sensors mainly include the protein kinase RNA-like endoplasmic reticulum kinase (PERK), the inositol-requiring enzyme (IRE1α), and activating transcription factor 6 (ATF6). Our previous study showed that ER stress was involved in the infection process of multiple pathogens in goat endometrial epithelial cells (gEECs) through different branches, such as LPS, *Brucella*, and *Trueperella pyogenes* [8,9,10]. Meanwhile, studies have reported that there is a close relationship between autophagy and the ER stress pathway, or that the two affect each other [11,12,13]. However, detailed in vitro data from ruminants on the role of autophagy and ER stress in the death induced by *S. aureus* are largely missing. Based on the previous research, we hypothesized that the autophagy and ER stress may be involved in the regulation of the cell apoptosis induced by *S. aureus*.

In the present study, we aimed to test this hypothesis by observing the effect of *S. aureus* on apoptosis, autophagy, and ER stress in gEECs, and further explored the relationship among them. This finding will provide new information to refine the mechanism of cell damage after *S. aureus* infections.

## 2. Materials and Methods

### 2.1. Regents and Antibodies

Dulbecco’s modified Eagles medium with Hams F-12 nutrient mixture (DMEM/F12, 1:1) was purchased from Hyclone (South Logan, UT, USA). Fetal bovine serum (FBS) was obtained from ZETA Life (Menlo Park, CA, USA). The antibody against β-actin was purchased from Tianjin Sungene Biotechnology Inc. (Tianjin, China). The antibodies against ATF-6, P-IRE1α, EIF2S1, P-EIF2S1, ATF-4, GRP78, and LC3I/II were purchased from Abcam (Cambridge, MA, USA). The antibody against caspase-3 was obtained from Abclonal (Wuhan, China). Antibodies against poly (ADP-ribose) polymerase 1 (PARP-1) and Bax were purchased from Proteintech (Wuhan, China). Likewise, 3-Methyladenine (3-MA), chloroquine (CQ), and 4-phenylbutyric acid (4-PBA) were obtained from Sigma-Aldrich (St. Louis, MO, USA). The LDH cytotoxicity assay kit was purchased from Promega (Madison, WI, USA).

### 2.2. Bacterial Strains and Growth Conditions

*S. aureus* (ACCC 01011) was a gift from the Veterinary Public Health and Livestock Product Safety Laboratory of Northwest A&F University. The frozen strains were revived and grown in Luria-Bertani (LB) solid medium at 37 °C for 12 h. After the colonies grew, the monoclonal colony was picked and grown in LB liquid medium at 37 °C on an orbital shaker (180 rpm/min) for 12 h. The bacterial growth was monitored by measuring the optical density at 600 nm (OD600). Tenfold serial dilutions of *S. aureus* were plated on LB for the bacterial enumeration. When indicated, *S. aureus* was resuspended in PBS to achieve the required density.

### 2.3. Cells and Infection

Immortalized goat endometrial epithelial cell lines were established and maintained in our laboratory. The cells were cultured in DMEM/F12 medium containing 10% FBS and 1% penicillin-streptomycin at 37 °C in a humidified 5% CO_2_ incubator. For the infection experiments, after back dilution, *S. aureus* was added to the cells at a multiplicity of infection (MOI) of 10 for up to 2 h or 4 h. For the inhibition experiments, the cells were pretreated 1 h before infection with 1 mM 4-PBA, 2.5 mM 3-MA, and 10 μM CQ, respectively.

### 2.4. Cytotoxicity Assay

The cytotoxicity was measured using a lactate dehydrogenase (LDH) assay. The release of the LDH into the culture supernatants was detected according to the manufacturer’s instructions. Briefly, the supernatants were collected after centrifuging for 1500× *g*/min for 5 min, 50 μL was transferred from all of the test and control wells to a new 96-well plate, then 50 μL of the CytoTox 96^®^ Reagent was added to each well and incubated at room temperature for 30 min away from the light. Finally, 50 μL of stop solution was added to each well, and the absorbance was recorded at 490 nm using the microplate reader. The following formula was used to compute the cytotoxicity:

Percent cytotoxicity = (Experimental LDH Release (OD490))/(Maximum LDH Release (OD490)) × 100%

### 2.5. Transmission Electron Microscopy (TEM)

The cells were fixed using 2.5% glutaraldehyde at room temperature, protected from light, and were subsequently fixed in a solution containing 1% osmium fixative. Then, the samples were dehydrated with a graded ethanol series, embedded in epoxy resin, and cut into ultrathin sections. The sections were then stained using 3% uranyl acetate–lead citrate cream, observed, and photographed using a transmission electron microscope.

### 2.6. Western Blot

The cells were washed twice with PBS, and 100 μL RIPA lysate was added to each well; they were subsequently lysed on ice for 30 min, and then centrifuged at 4 °C for 15 min at 12,000× *g*/min. Then, the supernatant was collected, and the protein concentration was assayed using a BCA protein concentration assay kit (Beyotime Biotechnology, Beijing, China). The proteins were run on an SDS-PAGE, then transferred to a PVDF membrane. After being blocked with 5% non-fat dry milk for 2 h at room temperature, the membranes were subsequently incubated with primary antibodies at 4 °C overnight. After three washes with TBST, the horseradish peroxidase (HRP)-conjugated secondary antibody was added and incubated at room temperature for 1 h. Finally, ECL luminescent solution was used to detect the bands of target proteins, and grayscale analysis was performed using Image J software (Version 1.8.0; Bethesda, Rockville, MD, USA).

### 2.7. Immunofluorescence Staining

After being infected with *S. aureus* for 4 h, the gEECs were washed with PBS three times and fixed with 4% paraformaldehyde for 20 min. Then, the cells were incubated with 0.3% Triton 100 for 5 min at room temperature, washed twice with PBS, followed by incubation with 3% BSA at room temperature for 2 h. The cells were then incubated with the ant-LC3II antibody at 4^o^C overnight. After washing three times with PBS, the cells were incubated with coralite488-conjugated goat anti-mouse IgG for 1 h. Laser scanning confocal microscopy was used to image the cells.

### 2.8. Statistical Analysis

The data were analyzed using GraphPad PrismTM 5 software (GraphPad Software Inc., La Jolla, CA, USA), one-way analysis of variance (ANOVA) was used for multiple-group comparisons, and a *t*-test was applied for two-group comparisons. The data are presented as the mean ± standard deviation (SD). Each experiment was repeated three times.

## 3. Results

### 3.1. S. aureus Induces gEECs Apoptosis

**Figure 1:**
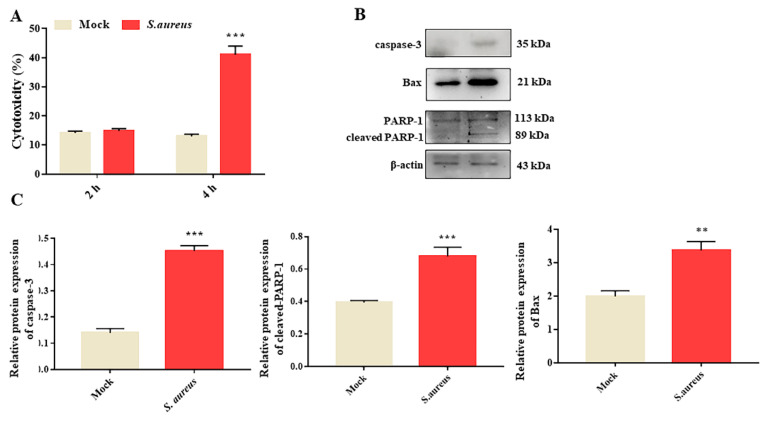
*S. aureus* induces gEECs apoptosis. (**A**) The cells’ cytotoxicity was measured by the LDH assay of *S. aureus*-infected (10 MOI) gEECs for 2 h and 4 h. (**B**) gEECs were infected with *S. aureus* for 4 h, and were subjected to immunoblot analysis for apoptosis-associated proteins. (**C**) The relative protein expression was calculated by comparing the ratio of the detected band with the β-actin levels. Each experiment was repeated three times. ** *p* < 0.01, *** *p* < 0.01 vs. the mock group.

In order to investigate the effect of *S. aureus* on gEECs cytotoxicity, cells were infected with *S. aureus* (10 MOI) for 2 and 4 h. Then, the culture supernatants were collected in order to detect the level of LDH released from the mock group cells (uninfected cells) and *S. aureus*-infected cells. As shown in Figure 1A, compared to the mock group, the LDH level had no significant changes after being infected for 2 h. At up to 4 h, *S. aureus* could induce significant cytotoxicity, and resulted in approximately a 40% release of LDH. Furthermore, we performed a Western blot assay to determine the effect of *S. aureus* on gEECs apoptosis at the indicated time (4 h). The results showed that, compared to the mock group, *S. aureus* markedly induced the protein expression of caspase-3, cleaved-PARP-1, and Bax (Figure 1B,C and Figure S1). These data indicate that *S. aureus* could induce gEECs apoptosis.

### 3.2. S. aureus Induces gEECs Autophagy

**Figure 2:**
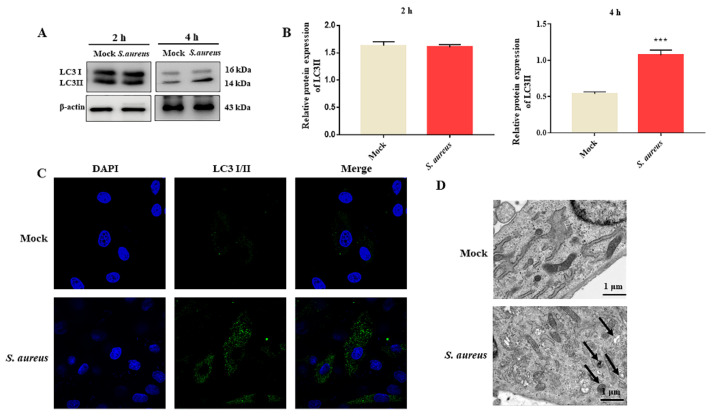
*S. aureus* induces gEECs autophagy. (**A**,**B**) Measurement of LC3II expression following *S. aureus* (10 MOI) infection for 2 h and 4 h using Western blot analysis. The results of the quantification are shown to the right. (**C**) gEECs were infected with *S. aureus* (10 MOI) for 4 h, and the LC3II-positive puncta were observed under the confocal microscope. Scale bars: 20 μm. (**D**) After being infected with *S. aureus* (10 MOI) for 4 h, the gEECs were analyzed by transmission electron microscopy (TEM). The typical autophagic structures are clearly visualized: black arrowheads depict the autophagosome. Scale bars: 1 μm. Each experiment was repeated three times. *** *p* < 0.001 vs. the mock group.

In order to determine whether *S. aureus* could induce gEECs autophagy, the expression of the autophagic marker protein (LC3) in *S. aureus*-infected gEECs at the indicated times was detected by Western blot assay. The results showed that, compared to the mock group cells, the expression of LC3 II had no significant changes after *S. aureus* infection at 2 h (Figure 2A and Figure S2); however, that of LC3II was significantly increased at 4 h (Figure 2B). In order to further confirm the effects of *S. aureus* infection on gEECs autophagy, confocal microscopy and a TEM assay were conducted. The results also revealed an increase in LC3II-positive puncta and autophagosomes with characteristic double-membrane-bound vesicles in the *S. aureus* group (Figure 2C,D). Taken together, the aggregate results suggested that *S. aureus* could induce gEECs autophagy.

### 3.3. Inhibition of Autophagy Rescued S. aureus-Induced gEECs Apoptosis

**Figure 3:**
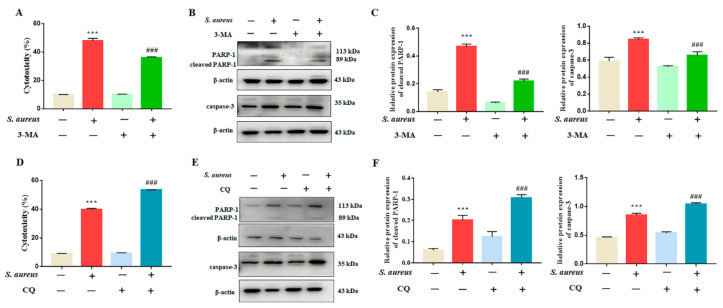
Effect of the inhibition of autophagy on gEECs apoptosis. (**A**,**D**) Percent cytotoxicity of gEECs infected with *S. aureus* (10 MOI) for 4 h, having been pretreated with the autophagy inhibitor 3-MA (2.5 mM) and CQ (10 μM), as determined using an LDH assay. (**B**,**C**,**E**,**F**) The protein expression of LC3II and caspase-3 was measured using Western blot analysis. Each experiment was repeated three times. *** *p* < 0.001 vs. the mock group, ### *p* < 0.001 vs. the *S. aureus* group.

Autophagy plays a key role in the regulation of the process of apoptosis, and further clarifies the role of autophagy in *S. aureus*-induced apoptosis in gEECs. The cells were pretreated with the early autophagy inhibitor 3-MA and the late autophagy inhibitor CQ for 1 h, then exposed to 10 MOI *S. aureus* for 4 h. The results showed that 3-MA rescued the cytotoxicity induced by *S. aureus* (Figure 3A), and decreased the expression levels of apoptosis-related proteins (caspase-3 and cleaved-PARP-1) increased by *S. aureus* (Figure 3B,C and Figure S3). However, CQ showed the opposite result of 3-MA, exacerbating *S. aureus*-induced apoptosis (Figure 3D–F and Figure S3). These data indicate that *S. aureus* could lead to the accumulation of autophagosomes in gEECs, which in turn promote gEECs apoptosis.

### 3.4. S. aureus Induces ER Stress in gEECs

**Figure 4:**
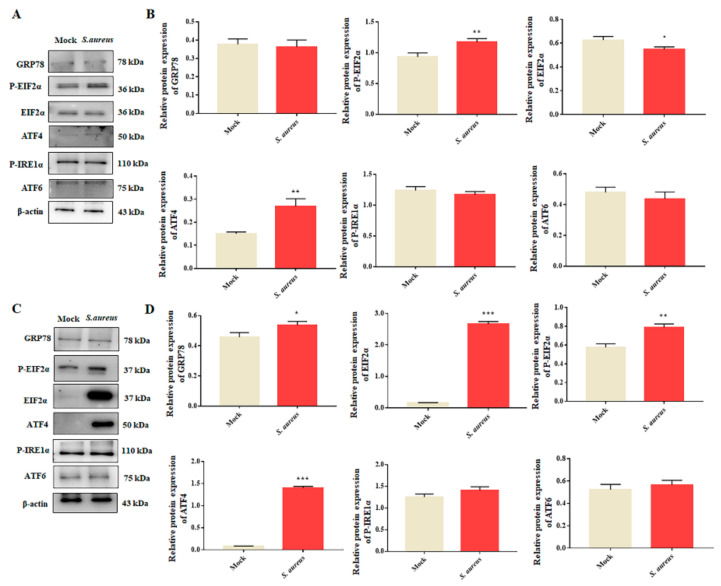
*S. aureus* infection induces ER stress in gEECs. (**A**,**B**) Western blot for ER stress-related proteins in whole-cell lysates from gEECs following infection with *S. aureus* (10 MOI) for 2 h, along with the protein abundance. (**C**,**D**) The expression of ER stress-related proteins after *S. aureus* (10 MOI) infection for 4 h, along with the protein abundance. Each experiment was repeated three times. * *p* < 0.05, ** *p* < 0.01 and *** *p* < 0.001 vs. the mock group.

In order to explore the effect of *S. aureus* on the ER stress pathway in gEECs, cells were infected with *S. aureus* of 10 MOI for 2 h or 4 h, and the expression levels of ER stress-related proteins (GRP78, P-EIF2α, EIF2α, P-IRE1α, ATF4, and ATF6) were measured by Western blot. As shown in Figure 4A,B and Figure S4, compared to the mock group, the expressions of P-EIF2α and ATF4 were significantly increased after *S. aureus* infection for 2 h. After infection for 4 h, the expressions of GRP78, EIF2α, P-EIF2α, and ATF4 were markedly increased, and those of ATF6 and P-IRE1α showed no significant changes (Figure 4C,D and Figure S4). These results suggest that *S. aureus* mainly activated ER stress in gEECs, mainly the EIF2α–ATF4 pathway.

### 3.5. Autophagy Rescues S. aureus-Induced gEECs Apoptosis via ER Stress

**Figure 5:**
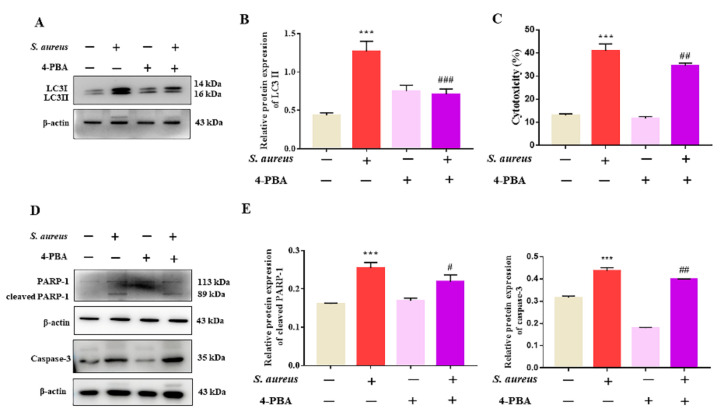
4-PBA attenuated *S. aureus*-induced gEECs autophagy and apoptosis. (**A**,**D**) gEECs were pretreated with 4-PBA (1 mM) and infected with *S. aureus* (10 MOI). The protein levels of LC3II, caspase-3, and cleaved-PARP-1 were quantified by Western blot. (**B**,**E**) The expressions of LC3II, caspase-3, and cleaved-PARP-1 were quantified by densitometry, and were normalized to β-actin. (**C**) LDH release was measured using a cytotoxicity LDH assay kit. Each experiment was repeated three times. *** *p* < 0.001 vs. the mock group, # *p* < 0.05, ## *p* < 0.01, and ### *p* < 0.001 vs. the *S. aureus* group.

In order to investigate the role of ER stress in *S. aureus*-induced gEECs autophagy, the cells were pretreated with the 4-PBA for 1 h, which was the inhibitor of ER stress, and then infected with *S. aureus*. After infection for 4 h, the expression of autophagy-related proteins was measured using Western blot. As shown in Figure 5A,B and Figure S5, the data showed that, compared to the *S. aureus*-infected cells, the 4-PBA treatment markedly decreased the expression of LC3II. Furthermore, we found that 4-PBA also significantly alleviated the gEECs’ cytotoxicity and decreased the expression of apoptosis-related proteins (caspase-3, cleaved-PARP-1) increased by *S. aureus* (Figure 5C–E and Figure S5). These data suggest that *S. aureus* induces gEECs apoptosis by regulating ER stress-mediated autophagy.

## 4. Discussion

*S. aureus* is considered to be a recognized zoonotic pathogen, which can infect many kinds of organs, such as the lungs, skin, eyes, and uterus. EECs, as an important physical barrier of the uterus, could also provide immune functions as immune cells [14,15,16]. Uncovering the mechanisms of EECs damage during *S. aureus* infection is essential in order to enhance endometrial tolerance. Reports have shown that *S. aureus* could cause multiple modes of cell death, including apoptosis, pyroptosis, and necrosis, which further exacerbate the infection process [17]. Recent studies have focused on the regulatory role of autophagy and ER stress in cell death [18,19], while their role in *S. aureus*-induced apoptosis has not been well elucidated. Hence, in the present study we aimed to explore the effect of *S. aureus* on gEECs autophagy, ER stress, and apoptosis, in order to try to provide more information on the mechanism of *S. aureus* infection.

LDH is a stable enzyme in cells, which could be released when cell membranes rupture and can be used to indicate cytotoxicity. Our results showed that *S. aureus* could cause significant cytotoxicity, and could induce gEECs apoptosis. Apart from apoptosis, excessive autophagosomes could be triggered after *S. aureus* infection. As the maker of autophagy, the expression of LC3II was upregulated. Reports showed that *S. aureus* could induce autophagy in mammary epithelial cells and immune cells [20,21], which was consistent with our results. Autophagy is a key process for the degradation of damaged components and organelles; the formation of autophagy mainly includes initiation, elongation, maturation, and degradation. Autophagy and apoptosis, as different forms of cell death, exist in interaction with each other. In general, autophagy helps cells to handle waste and adjust themselves to an adapting real-time milieu; however, excessive autophagy may result in cell death. Reports have shown that autophagy can act as a superior regulator of apoptosis, thus affecting cell death [22,23]. Furthermore, autophagy could promote cell survival via the inhibition of cell apoptosis. However, the relationship between the gEECs apoptosis and autophagy induced by *S. aureus* has not been experimentally revealed. We further explored the mechanism by using the early autophagic inhibitor 3-MA, which mainly inhibits the formation of autophagosomes, and the late autophagic inhibitor CQ, which mainly inhibits the fusion of autophagosomes and lysosomes. We found that 3-MA alleviated *S. aureus*-induced apoptosis, while CQ enhanced the apoptosis, suggesting that *S. aureus* facilitated the accumulation of autophagosomes in gEECs, ultimately promoting apoptosis. 

Numerous studies have shown that ER stress was involved in several physiological and pathological conditions, including apoptosis, autophagy, and the immune response [24,25]. Different stressors activate different UPR pathways; in addition, the same stressor may activate different UPR pathways in different tissues or cells. Kumar reported that the IRE1/XBP-1 pathway regulated the retinal innate immune response in *S. aureus*-induced endophthalmitis [26]. *S. aureus* enterotoxin B increased the expression of GRP78 and P-EIF2α in human epithelial cells, which are strongly associated with inflammation [27]. Li with colleagues showed that the EIF2α–ATF4 pathway was activated in *S. aureus*-infected HNEpCs [28]. Our findings supported the supposition that *S. aureus* induced the EIF2α–ATF4 branch of ER stress, but not the other classical ER stress sensors. After infection of 2 h, the expressions of ATF4 and P-EIF2α were increased; however, that of GRP78 was not changed. The results indicate that other cellular responses were activated early in the *S. aureus* infection of the gEECs. The EIF2α showed a trend of decreasing and then increasing expression, which may be an adaptive regulation of gEECs in response to *S. aureus* infection. Amino acid starvation acts as an activator in the integrative stress response (ISG), and could induce EIF2α–ATF4 pathway expression [29], and reports have shown that *S. aureus* infection can lead to amino acid starvation, which in turn activates autophagy [30]. The definition of the relationship between EIF2α–ATF4 and autophagy will be further investigated. Furthermore, we initially explored the effects of ER stress on autophagy and apoptosis. The ER stress inhibitor 4-PBA was used to determine this effect, and it was found that the inhibition of ER stress could decrease the expression of LC3II and apoptosis.

## 5. Conclusions

In summary, we reported here that *S. aureus* could induce gEECs autophagy and ER stress. The accumulation of autophagosomes contributed to *S. aureus*-induced gEECs apoptosis, and the ER stress pathway was involved in the regulation of autophagy. These findings uncover an important mechanism of gEECs death after *S. aureus* infection, and could provide new insight into the therapeutic target of endometrial cell injury.

## Data Availability

The data presented in this study are available upon request to the corresponding author.

## Supplementary Materials

The following are available online at https://www.mdpi.com/article/10.3390/ani12060711/s1, Figure S1: the original western blot figures of apoptosis-related proteins after *S. aureus* infection. Figure S2: the original western blot figures of autophagy-related proteins after *S. aureus* infection. Figure S3: the original western blot figures of apoptosis-related proteins in 3-MA or CQ-pretreated cells. Figure S4: the original western blot figures of ER stress-related proteins after *S. aureus* infection. Figure S5: the original western blot figures of proteins of apoptosis and autophagy in 4-PBA-pretreated cells.
