# Supplementary figures and images for: Staphylococcus aureus Induces Goat Endometrial Epithelial Cells Apoptosis via the Autophagy and Endoplasmic Reticulum Stress Pathway
Source: Animals (Basel). 2022 Mar 11;12(6):711. doi: 10.3390/ani12060711 (PMC8944437; doi:10.3390/ani12060711)

Figure S1

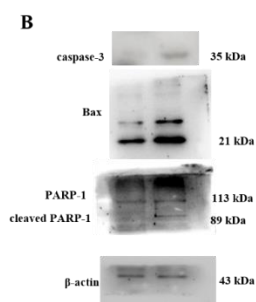

Figure S2

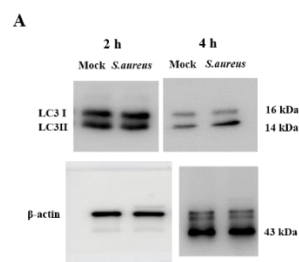

Figure S3

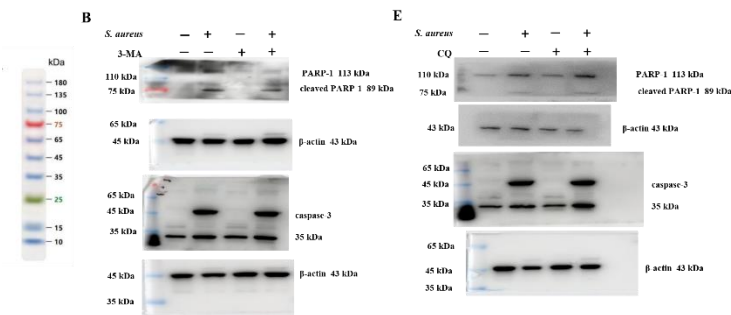

Figure S4

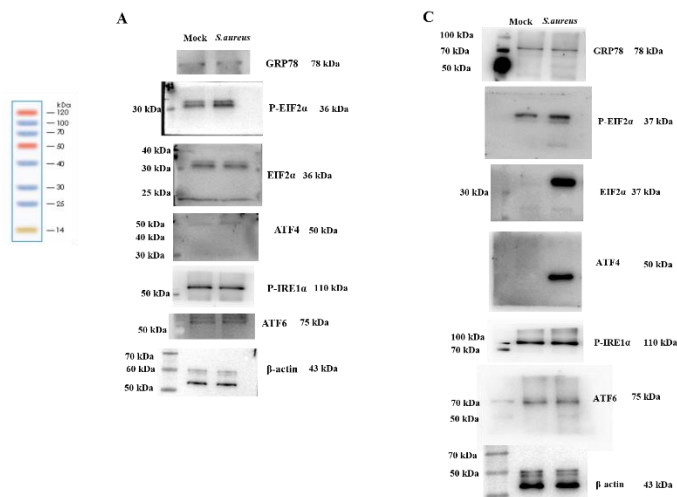

Figure S5

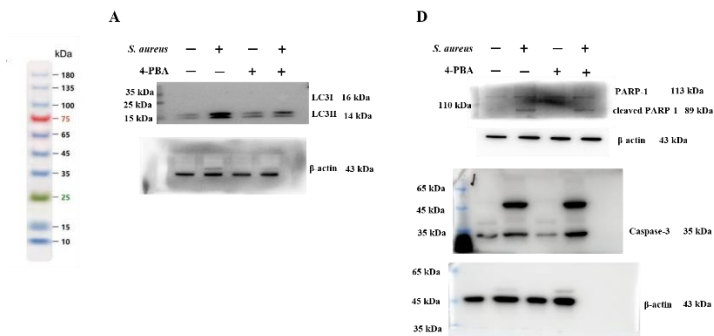

Supplement: Supplementary file 1 [file animals-12-00711-s001.zip › animals-1603324-supplementary.pdf]
